# Low Silicon and Better Pasture Feeding Quality: Uncovering Genetic Diversity in Russian Wildrye (*Psathyrostachys juncea*)

**DOI:** 10.3390/life16040562

**Published:** 2026-03-30

**Authors:** Svetlana Dashkevich, Maral Utebayev, Nadezhda Filippova, Oksana Kradetskaya, Irina Chilimova, Irina Rukavitsina, Gulmira Khassanova, Satyvaldy Jatayev, Yuri Shavrukov

**Affiliations:** 1 A.I. Barayev Research and Production Centre of Grain Farming, Shortandy 021601, Kazakhstan; phytochem@yandex.ru (M.U.); filippova-nady@mail.ru (N.F.); oksana_cwr@mail.ru (O.K.); coronela@mail.ru (I.C.); irukavitsina@mail.ru (I.R.); 2 Institute of Agronomy and Forestry, S. Seifullin Kazakh AgroTechnical Research University, Astana 010000, Kazakhstan; g.hasanova@kazatu.edu.kz (G.K.); s.jatayev@kazatu.edu.kz (S.J.); 3 College of Science and Engineering, Biological Sciences, Flinders University, Adelaide, SA 5042, Australia

**Keywords:** biochemical composition, genetic diversity, germplasm collection, hybrids, pasture biomass quality, pasture crop breeding, *Psathyrostachys juncea*, Russian wildrye, silicon

## Abstract

In this study, 72 genetically diverse accessions of *Psathyrostachys juncea* from a germplasm collection were evaluated for silicon content, biochemical composition and nutritional value in pasture biomass for grazing feed in Northern Kazakhstan in 2024 and 2025. High-quality biomass and low silicon are the most important traits for *P. juncea* pasture. In the studied germplasm collection, the average silicon content in leaves was 2.59%, ranging from 1.45% to 4.11%. All studied accessions of *P. juncea* were split into two clusters based on biochemical analyses. Cluster B with preferable genotypes had significantly lower silicon content, crude fibre, neutral detergent fibre and hemicellulose, but higher crude protein content compared to cluster A. The six best genotypes with close to or less than 2% silicon and with high nutritional value in pasture biomass content were selected from cluster B for hybridization and further breeding. Low silicon content in leaves was confirmed in most of the hybrids, similar to parents and significantly less than other genotypes in the germplasm collection. Strong negative heterosis values were identified in all hybrids for acid detergent fibre and lignin, showing a reduction in undesired traits for biomass pasture quality. A strong negative correlation was found between the content of crude protein and fibre (*r* = −0.71), whereas neutral and acid detergent fibre content had a strong positive correlation (*r* = 0.78). The most promising hybrids with the combined traits of low silicon accumulation and high-quality pasture biomass were selected for further breeding and production of new perspective cultivars of *P. juncea* for pastures with perennial forage plant species.

## 1. Introduction

Russian wildrye [*Psathyrostachys juncea* (Fisch.) Nevski] (formerly, *Elymus junceus* Fisch.) is a promising perennial forage crop belonging to the family Poaceae. This plant species is widespread in North America, including in central United States [1,2], Canada [3] and Alaska; in Asian countries like Mongolia, Afghanistan, western China [4] and Tibet; and in the central and northern regions of Kazakhstan [5], Kyrgyzstan, and in Russia, including southern regions of Western and Eastern Siberia [6,7], the Volga region and Yakutia [8].

In 1934, Russian botanist, Sergiy A. Nevski, in the fundamental ‘Flora of the USSR’ [9], described and subdivided the genus *Elymus* L. into five independent species based on differences in the structure of the flower, spike, number of florets in the spike and some other traits, and the species *E. junceus* Fisch. was assigned to the genus *Psathyrostachys* Nevski. The author explained that the Latin name comes from the Greek words “psathyros”—brittle and “stachys”—ear or spike, as the axis of the ear can be broken into segments.

In Kazakhstan, *Psathyrostachys juncea* with the Kazakh name ‘Tarlau’ was introduced into cultivation in 1948, in Alma-Ata region on the Bozoy Experimental Station, where in 1970 the first cv. Bozoisky was produced and distributed widely throughout the world [10]. In the USA and Canada, the cultivars Vinall, Sawki, Mankota, Cabree and Bozoisky II (or Bozoisky Edit) [2,11,12,13] are well-known, as well as some other cultivars: Majak, Swift, Tetracan and Tom [14,15]. Field trials in Utah, Idaho, and Wyoming showed that cv. Bozoisky, introduced from Kazakhstan, had the highest productivity and salt tolerance [10]. Modern breeding programs for *P. juncea* in Kazakhstan have successfully produced two new cultivars: Shortandinsky and more recently, Faradiz [16].

A specific trait of *P. juncea* is the uniform distribution of forage biomass across successive cuttings. After grazing or cutting (before the heading stage), only genotypes with a strong capacity for re-growing have the potential for recovery and continued future production [17]. *P. juncea* is a valuable forage crop providing a good harvest and high nutrient quality of pasture biomass in early- and late-spring periods [18].

High quality in pasture biomass is determined by the presence of key nutrients and their various characteristics or traits, most important of which are crude protein, fat, ash, fibre and carbohydrates. Based on published reports, plants of *P. juncea* have valuable and very-high-quality pasture biomass compared to other grasses. For example, crude protein was recorded at 14.84% in *P. juncea* [19], similar to the 10–17.46% crude protein determined in various grasses [20]. However, contents of crude fibre and fat, 21.89% and 2.36%, respectively, were slightly lower than the 29–30.4% and 2.8–3.7%, respectively, found in other pasture grasses [21].

Three other components, neutral detergent fibre (NDF), acid detergent fibre (ADF) and acid detergent lignin (ADL), represent indicators for digestion rate and consumption by animals, feed quality and digestibility, and cell lignification, respectively [22]. In grasses like orchard (*Dactylis glomerata* L.), tall fescue (*Festuca arundinacea* Schreb.) and bromegrass (*Bromus inermis* Leyss.), NDF varied between 51.9 and 57.7%, whereas ADF and ADL were reported as 29.9–33.9% and 2.9–4.1%, respectively [22,23].

Regarding the polysaccharides cellulose and hemicellulose, their content varies depending on the plant species, developmental stage and tissue, with the cell wall containing on average 40–60% cellulose and 20–40% hemicellulose [24]. However, cellulose should be around 21–30% of dry matter and hemicellulose about 10–20% [22,23,25].

In Kazakhstan, in the natural grasslands of dry steppes, *P. juncea* makes up a significant part of pastures [26]. This plant species cross-pollinates and pollen is carried by the wind. Flowering time depends mostly on weather conditions; flowering is delayed when soil and air humidity are high but is stimulated by hot weather [27]. With sufficient rainfall, *P. juncea* will grow and develop well, whereas without moisture, development stalls and the plants may turn yellow and dry out. However, with even light rainfall, *P. juncea* plants re-grow rapidly, turn green and produce new vegetative shoots.

Adaptation to low temperatures allows this crop to grow in the earliest spring periods, which ensures its early maturity [27]. It was noted that *P. juncea* is a typical pasture crop but poorly adapted for cutting and silage or hay preparation. This is because all leaves are located in a basal rosette rather than along stems, and it is therefore difficult to collect biomass mechanically after cutting [28,29].

One of the problems limiting the spread of forage crops, like *P. juncea*, is the presence of silicon imparting rigidity to plants [30]. Cereals typically have high silicon content (more than 4% of dry weight) [31]. It was shown that plant defence responses to fungi are faster and more efficient in the presence of silicic acid in wheat [32] and *Arabidopsis* [33], suggesting that Si acts as a biochemical mediator. Additionally, it was reported that water-soluble silicon inhibits the activity of cellulases and other digestive enzymes, while insoluble forms remain chemically inert [34,35]. In both laboratory and field conditions, increased silicon occurs in response to herbivory. For example, voles fed plant matter high in silicon as a result developed slower and had higher mortality compared to controls [36]. Laboratory studies with captive animals have convincingly shown that voles and rabbits began to eat less grass with a high content of silicon [37,38,39].

Additionally, silicon spines and other sharp teeth-like structures known as phytoliths, generally only visible under a microscope, make the leaf blade of *P. juncea* abrasive, which can also injure the tongues of grazing animals. Silicified cells, in addition to leaf blades and their edges, were also observed in the epidermis and vascular tissues of the stem, sheath and leaf [40]. Similar results were obtained in rice, which is a typical silicon-accumulating plant species [41]. Silicon can account for up to 10% of the dry weight of rice shoots, and this is several folds higher than essential macronutrients such as nitrogen, phosphorous and potassium. Silicon is deposited under the leaf cuticle, forming a thin film or layer called a ‘cuticle-Si double layer’, which increases the strength and hardness of the cell wall. These Si deposits protect plants from a variety of abiotic and biotic stresses [42] but high silicon can be very destructive for grazing animals and can reduce the quality of forage crops, including *P. juncea*.

Currently, two major genes, *Lsi1* and *Lsi2*, have been identified and described, encoding the influx and efflux of silicon, respectively, resulting in transportation and accumulation of Si in roots, shoots and leaves in rice plants [43], pumpkin and other dicotyledonous plant species [44]. High silicon accumulation associated with the activity of *Lsi1* and *Lsi2* genes has been confirmed in ryegrass [45], bamboo (*Phyllostachys edulis*) [46] and other plant species [47].

Based on our analyses of the germplasm collection of Russian wildrye, *Psathyrostachys juncea*, the hypothesis of this study was to combine low silicon content and improved quality of pasture biomass in the developed breeding lines. Therefore, the aim was to assess the genetic diversity of silicon content and key pasture biomass quality traits within accessions and make a proven conclusion about the most promising hybrids.

## 2. Materials and Methods

### 2.1. Plant Material and Hybridization

**Figure 1:**
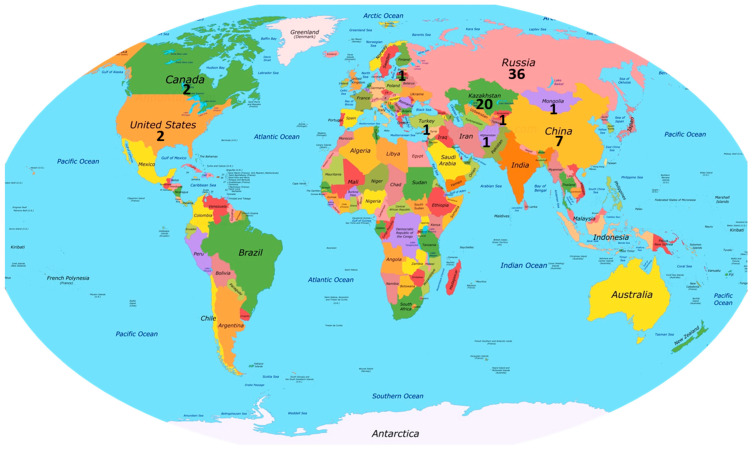
Geographic distribution of 72 studied accessions of *Psathyrostachys juncea.* Number of studied accessions is indicated under name of corresponding country.

Seeds of all 72 accessions of Russian wildrye, *Psathyrostachys juncea* (Fisch.) Nevski, used in this study were received from N.I.Vavilov Research Institute of Plant Industry (VIR), St-Petersburg, Russia, in 2019. The full list of accessions and their origin is present in Supplementary Table S1. The geographic distribution of studied accessions included 36 from Russia, 20 from Kazakhstan, 7 from China, 2 each from Canada and the USA, and one each from Afghanistan, Estonia, Kyrgyzstan, Mongolia and Türkiye (Figure 1).

**Figure 2:**
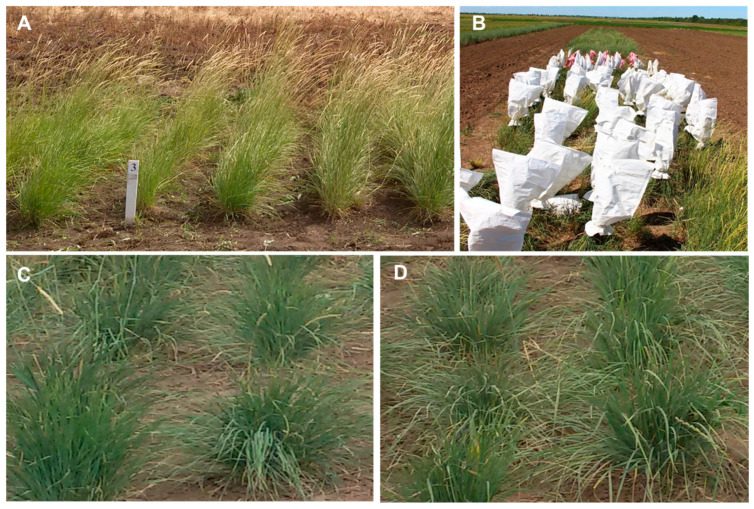
An example of studied *P. juncea* accessions in the research field (**A**); inflorescence isolation by paper bags (**B**); hybrids KL-1804 (**C**); and hybrids KL-1805 (**D**).

After initial evaluation, several of the most promising accessions were selected for hybridization. One inflorescence from each maternal and paternal form was isolated together in a paper bag for natural cross-pollination. Plants of *P. juncea* species are strictly cross-pollinated [18], so all seeds produced will be hybrids since self-pollination does not occur in regular conditions. An example of the studied *P. juncea* accessions in the research field, the paper bag isolation on their inflorescence and some of the resulting hybrids is presented in Figure 2.

### 2.2. Field Growth and Biomass of Cut Samplings

The field trial was carried out at the A.I.Barayev Research and Production Centre of Grain Farming (RPCGF), Shortandy (Kazakhstan), at an altitude of 357 m above sea level, 51°40′ N 71°01′ E. Soil was carbonated chestnut chernozem with 0.3% total nitrogen, 0.1% phosphorous and up to 5% carbonate. Slightly alkaline soil pH was recorded, ranging between 7.6 and 7.9. Sowing was carried out in the spring, on bare fallow, with previous autumn tillage by a deep cultivator. Crop care consisted of inter-row cultivation and manual weeding. Fertilisers, fungicides or insecticides were not applied.

In 2021, seeds of 72 accessions from the germplasm collection were sown manually in a research field in single-row plots, 3 m in length and with 0.6 m between rows, with a plot area of 1.8 m^2^ and density of 35–37 plants per m^2^ in each plot. Each genotype has four replicated plots with a completely randomised design (CRD) in the field trial. For analysis of hybrids, seeds were sown in similar plots, side-by-side with the parents for better visual comparisons. After 3–4 years of plant growth after sowing, all 72 accessions and their hybrids were evaluated over two years: 2024 and 2025. The precipitation was different in these years, whereas temperature was relatively stable (Table 1). From April to August, total precipitation was 214.4% higher than the past average in 2024 but 56.1% lower in 2025, which was reflected in silicon accumulation and other traits studied.

Cutting of pasture biomass, imitating grazing, was carried out twice, once in the middle of May and again three weeks later, at the beginning of June. However, for biochemical analyses, only first cuts were used in both years and treated as separate samples for pasture analysis. At the cutting stages, plants of *P. juncea* were 40–45 cm in height prior to the appearance of reproductive shoots. The biomass of each cut plot was divided into three similar parts that were treated as three biological replicates for each of the studied genotypes. Each replicate was dried in a 60 °C incubator over five days until completely dry before being crushed and ground in a Lab-mill Perten-3100 (Perkin Elmer, Shelton, CT, USA) with a 0.8 mm sieve. Dried, ground samples were kept in a desiccator with silica gel until further biochemical analyses.

### 2.3. Silicon Determination

Quantitative determination of silicon compounds was carried out following a published protocol [48] with minor modifications. Dried and ground samples were ashed in ceramic crucibles (cups) at a temperature of 550–650 °C in a muffle oven. The ash was dissolved in 10 mL of a mixture of hydrochloric and nitric acids (1:3) with heating at 100 °C for 1 min and subsequent filtration using Whatman ashless filter paper. The filter paper was then rinsed and ashed in clean crucibles at the same temperature of 550–650 °C and weighed after cooling for quantification of Si content.

### 2.4. Determination of Crude Protein and Fibre

Crude protein content was determined in a UDK 139 Semi-Automatic Kjeldahl Distillation Unit (VEIP Scientifica, Usmate, Italy) [49]. The method consists of destroying organic matter with sulfuric acid in the presence of a catalyst, adding an alkali and absorbing the released ammonia, followed by titration. The result is expressed as nitrogen re-calculated for crude protein by multiplying by a coefficient of 6.25.

Crude fibre measurement in the study was carried out in accordance with [50], and is based on the oxidation, destruction and dissolution of various chemical compounds that make up plants with acids and alkalis. In this case, the fibre is the only component not dissolved, so it can then be filtered out and weighed. These analyses were carried out using a 12-place fully automatic system, Fibretherm FT12 (Gerhardt, Oberdollendorf, Germany).

### 2.5. Determination of Crude Fat and Ash

Crude fat content was measured using a ST243 Soxtec device (Foss, Hilleroed, Denmark). It was based on a fully automatic system in extraction and hydrolysis units with a solvent and optimal heat transfer to extraction cups with the analysed sample and final quality control [51]. Crude ash was determined as the mass of the residue after combustion and subsequent calcination of the sample was carried out in ceramic crucibles in a muffle oven at 550–650 °C for 15 min with subsequent cooling [52].

### 2.6. Determination of Structural Carbohydrates

The content of structural carbohydrates was determined using neutral and acidic detergents as described earlier [53,54,55]. All these analyses were carried out using a 12-place fully automatic system Fibretherm FT12 (Gerhardt, Oberdollendorf, Germany). Neutral detergent fibre (NDF) and acid detergent fibre (ADF) refer to the fraction of substances that are not dissolved with neutral or acid detergent solution, respectively [56,57]. Acid detergent lignin (ADL) was determined by dissolving cellulose with sulfuric acid in the ADF residue [58]. Cellulose content was calculated as the difference between ADF and ADL concentrations, and hemicellulose content was assessed as the difference between NDF and ADF concentrations.

### 2.7. Dominance and Heterosis

Data on hybrid plants in the first generation were used to determine true heterosis (H_true_), the ability of F_1_ hybrids to surpass the best of the parent for a studied trait [59], and degree of dominance (Hp) was calculated according to [60].

### 2.8. Statistical Treatments

Excel 365 (Microsoft) and IBM software packages SPSS, version 25.0.0.0 were used to calculate means and standard errors. To determine significant differences between genotype means in pair groups for the biochemical analyses, a *t*-test was used for pairwise comparisons with equal or unequal variances of samples. The null hypothesis indicated no difference between studied variances. The level of significance was determined via *t*-test and indicated by asterisks. Clustering for silicon and nutrient contents among 72 accessions of *P. juncea* was carried out based on Ward’s method of Minimum variance with Euclidean distance between clusters using computer software Statistica, version 6.0 (StatSoft Inc., Fort Lauderdale, FL, USA). Pearson correlation coefficient (*r*) was determined between all variable measurement results using Excel 365 (Microsoft) software. Three biological replicates and two technical repeats were used for biochemical analysis of each sample.

## 3. Results

### 3.1. Analysis of Genetic Diversity for Biomass Quality Traits in the Russian Wildrye Collection

The levels of silicon accumulation and pasture biomass quality of *P. juncea* genotypes from the collection were studied in the conditions of Northern Kazakhstan. As a result of our analyses for both 2024 and 2025, average values as well as minimum to maximum ranges were determined for each trait, including accumulation of silicon, biochemical composition and nutritional value of pasture biomass for animal feeding (Table 2).

**Figure 3:**
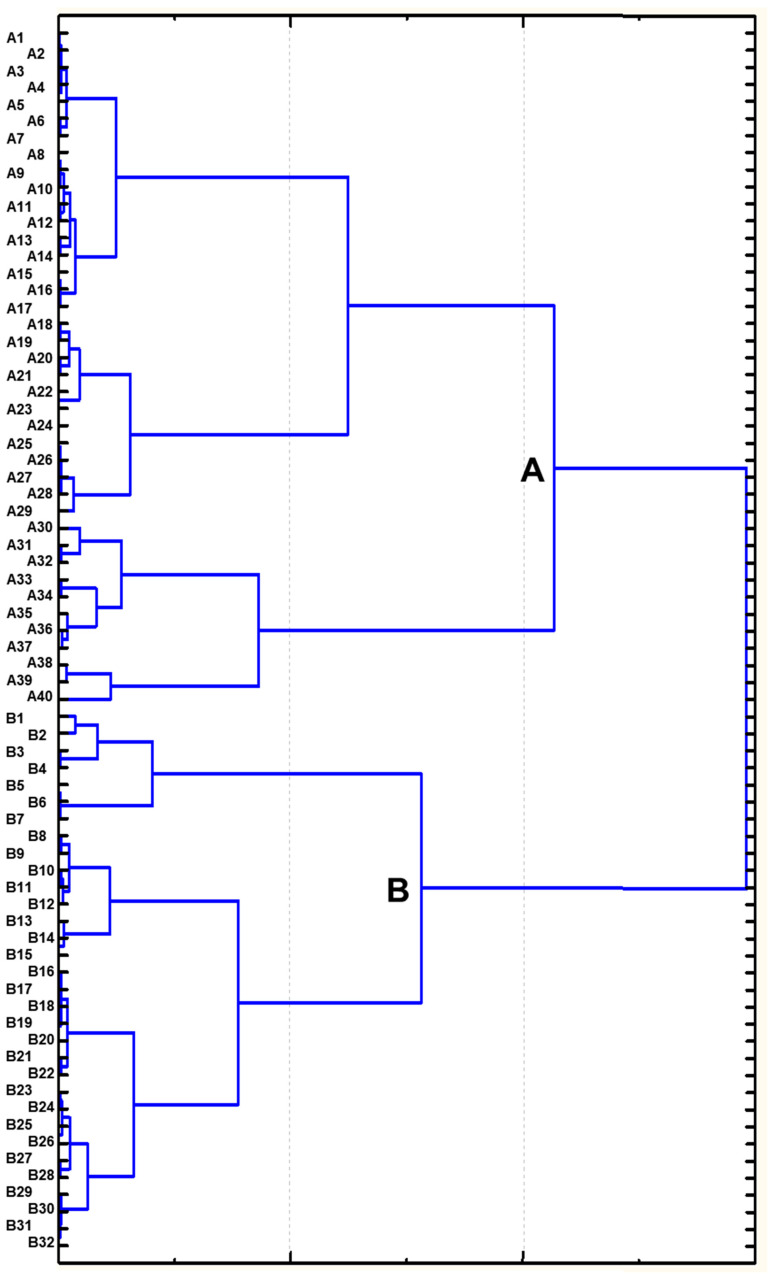
Cluster analysis of 72 accessions of *Psathyrostachys juncea* for silicon content and nutritional feed quality traits of pasture biomass, Northern Kazakhstan, 2024–2025.

These results were reflected in the clustering analysis based on silicon accumulation and all other traits for nutritional value in pasture biomass, where all 72 *P. juncea* genotypes were distributed in two clusters, A and B, with high and low Si content, respectively (Figure 3). A full list, distribution in two clusters, data for silicon and all nutritional feed quality traits, and results of *t*-test statistical analyses are present in Supplementary Table S2.

Cluster A included 40 accessions with higher silicon content averaging 2.77% and varying between 1.73% and 4.11%. In contrast, cluster B comprised 32 lower Si genotypes with a range between 1.45 and 3.42% and an average of 2.37%. The difference in silicon content in both clusters was highly significant. Crude protein was slightly higher in cluster B but still significantly different from cluster A (*p* < 0.05). A more significant difference was found for crude fibre, which was lower in cluster B (*p* < 0.01). In further trait analysis for nutritional pasture quality, only NDF and hemicellulose content were also significantly lower in cluster B (*p* < 0.05), whereas differences were insignificant in all other traits studied (Table 2, Figure 3, Supplementary Table S2).

### 3.2. Six Best Selected Accessions of Russian Wildrye from Cluster B with Low Silicon Content

Thirty-two accessions of *P. juncea* from cluster B were assessed and the best six with low silicon content were selected for further analyses and hybridisation. These results are present in Table 3 with the comparison to cv. Shortandinsky used as a standard (check). The first five genotypes (B1-B5) were located together in one sub-clade, whereas the last selected accession (B8) belonged to the next sub-clade. Comparison with the standard cultivar indicated no significant difference for silicon accumulation with the average of six accessions in 2024. However, the difference was highly significant in 2025 and for the average over both years combined (Table 3).

### 3.3. Hybrid Analysis, Heterosis and Degree of Dominance

Hybrids were evaluated for heterosis compared to their parents (Figure 2C,D; Table 4). For silicon, all hybrids showed positive levels of true heterosis. However, silicon content in hybrids KL-1804 and KL-1805 remained very low and below 2% (Table 4A). In hybrids KL-1808, the heterosis was high due to 2.11% of silicon accumulation. In contrast, hybrid KL-1809 accumulated lower silicon (1.77%) resulting in a much smaller heterosis (Table 4B). The last three hybrids showed significantly higher Si content (above 2%) compared to maternal parent K-43934 associated with high heterosis (Table 4C). The highest crude protein content in the pasture biomass was demonstrated in the three hybrids, KL-1813, KL-1808 and KL-1805, but it was not significantly different from the best corresponding parent. Therefore, a positive true heterosis effect at the level of overdominance for crude protein content was recorded in these hybrids but it was not so high: KL-1813, 5.28%; KL-1808, 1.50%; and KL-1809, 1.15% (Table 4).

The levels of the most important parameters, such as crude fibre, acid detergent fibre and acid detergent lignin, must be reduced for improvement of forage quality. The crude fibre content in pasture biomass was reduced in three hybrid combinations of hybrids KL-1805, KL-1816 and KL-1820, but it was statistically insignificant and yielding a heterosis effect in a relatively low range of −0.40 to −3.34% (Table 4).

All hybrids with the exception of KL-1813 showed decreased ADF content compared to the best corresponding parents, with significant and highly significant differences, and the heterosis effect ranged from −8.5 to −18.8% with a high level of dominance over the parental forms. For ADL content, all hybrids showed overdominance in the frame between −39.6 and −78.2%, confirmed by very high significant differences (Table 4).

### 3.4. Correlations

A very strong negative correlation was identified between crude protein and fibre with *r* = −0.71 but, in contrast, a similar very strong but positive correlation (*r* = 0.78) was found between NDF and ADF (Table 5). A less strong but still high level of correlation was revealed in the analysis between contents of silicon and crude ash (*r* = 0.63); crude fibre and ADF (*r* = 0.57); and between NDF and hemicellulose (*r* = 0.54), whereas crude protein and ADF had a similar but negative correlation (*r* = −0.52). All other correlations were less significant or not significant at all between studied traits for silicon and pasture biomass quality (Table 5).

## 4. Discussion

### 4.1. Silicon Content

The quality of forage crops is determined by their nutritional value, which directly depends on climatic conditions and plant maturity, as well as on the concentration of chemical components in plant tissues [61,62,63]. Silicon is well-known as an important element for cell wall structure, making plants more resistant to pathogens and pests. However, high silicon content reduces forage quality, having a strong negative impact on digestibility in pasture animals [64,65,66]. Silicon accumulation in plants very much depends on weather conditions and genotypes. In the current study, for example, in six selected parents, silicon content in pasture biomass was stable in both years, whereas in standard cv. Shortandinsky, silicon content was similar in the wetter conditions of 2024, but it was significantly higher in dry year of 2025, resulting in significant differences in total average for the two years (Table 3).

### 4.2. Crude Protein and Crude Fibre Content

Pasture biomass quality traits and silicon content were studied in the entire germplasm collection and also in two clusters: A and B (Table 2 and Figure 3). The pasture forage biomass of *P. juncea* consists of basal leaves (15–45 cm) and short vegetative shoots with long leaf blades [27]. In the current study, crude protein in pasture biomass across the genotypes of the collection was 20.71% on average (varying 14.84–23.69%), and these results were even higher compared to those published earlier: 15–20% [67] and 17.08–20.14% [68] of crude protein in other *P. juncea* accessions. This provides further confirmation that the identified genotypes in the studied germplasm collection contained a high level of crude protein. The second important component of pasture biomass quality is crude fibre, which has to be in balance with crude protein. In the current study, crude fibre in *P. juncea* germplasm accessions was in the range 21.98–27.37%, very similar to other reports [67,68].

Additionally, crude protein in cluster B was 21.16%, which is not very high but still significantly higher than the 20.34% in cluster A. Therefore, plants of *P. juncea* from cluster B could provide feed for animals with a higher protein content, which is preferable for improving their overall productivity. However, higher levels of protein require a balance with other nutrients, in particular in crude fibre as it was present at 23.63% in cluster B, to avoid animal metabolic disorders. At the same time, the pasture biomass from genotypes in cluster A was acceptable but not in optimal balance between the slightly lower crude protein (20.34%) and higher crude fibre (24.49%) with reduced total nutritional value in cluster A (Table 2).

### 4.3. Crude Ash and Crude Fat Content

Crude ash is representative of cell wall components, and a high content of crude ash is linked with low digestibility by animals. In the current study, the averaged 10.68% crude ash showed no significant difference between clusters A and B (Table 2). Our presented results were similar to crude ash in birdsfoot trefoil (*Lotus corniculatus* L.) with 8.1–10.0% of crude ash [69], and this is good indicator. Crude fat is the primary energy source improving palatability for feeding ruminants, and higher crude fat is preferred for pasture quality. In the current study, the average crude fat was 2.64%, without significant difference between clusters A and B, and it was slightly higher than the 1.7–2.5% published for *P. juncea* plants earlier [68].

### 4.4. Neutral Detergent Fibre (NDF) Content

In previous reports on pasture biomass quality in *P. juncea* genotypes, it was found that in the group with low dry matter digestibility, all samples had low crude protein content and high NDF content [70]. Therefore, the reduction in NDF is linked with better forage biomass quality. In the current study, in pasture biomass of *P. juncea* accessions in the studied germplasm collection, the average NDF content was 46.05%, and it was slightly but still significantly lower in cluster B (45.41%) compared to cluster A (46.60%). Nevertheless, the presented NDF content in both clusters was lower than those published earlier for forage grasses [22,23], and it was optimal for pasture plant biomass, indicating good cellular structure in these studied genotypes and therefore better resilience and longevity in pastures.

### 4.5. Acid Detergent Fibre (ADF) and Lignin (ADL) Content

ADF and ADL content are key indicators of feed quality and digestibility, which ideally should not be very high [71,72]. In the current study, ADF content was moderate, ranging from 19.07% to 28.38%, with an average of 21.95%, which may limit nutritional value. In contrast, levels of ADL in the studied genotypes were low, 1.63–5.13%, and 2.58% on average, indicative of weak cell lignification and, therefore, high feed quality. Both ADF and ADL did not differ in clusters A and B (Table 2), and they were at levels similar to those determined in forage grasses previously [22,23].

### 4.6. Cellulose and Hemicellulose Content

In the current study, the content of hemicellulose was 20.54–27.52%, with an average of 24%, and it was significantly lower (23.64%) in cluster B compared to cluster A with 24.29% hemicellulose. In contrast, no difference was found for cellulose content between the two clusters with an average of 19.46% (ranging, 16.01–24.95%) in the entire studied germplasm collection (Table 2). Our current results indicated smaller values of cellulose and hemicellulose compared to those published for pasture grasses with 30.0% and 28.2%, respectively [73].

### 4.7. Association Between Silicon and Biomass Nutrient Value

In the presented results, six *P. juncea* accessions from cluster B (Figure 3) with the lowest silicon content of 1.45–2.06% and averaging 1.71% were selected for hybridization and further analyses (Table 3). However, three genotypes, K-43934, K-46752 and K-46754, had decreased nutritional value due to their higher crude fibre content. Three other selected *P. juncea* lines, K-1731, K-36812 and K-40193, showed lower than average crude fibre content. This is an important conclusion indicating that low silicon had no strong association with nutrient value of pasture biomass (Table 3 and Supplementary Table S2).

### 4.8. Hybrid Analysis

The major aim of the current research was to reduce silicon content in pasture biomass and select the best hybrids considered to show results indicating low silicon. Despite positive heterosis and slightly increased silicon content in hybrids, their level remained very low and below 2% in three hybrids, and low–moderate in four other hybrids, ranging 2.06–2.33% (Table 4).

Improving feed quality requires the reduction in three important traits: crude fibre, ADF and ADL. For crude fibre, a negative heterosis effect and reducing the content (between −0.4 and −3.3%) was obtained in three hybrid combinations: KL-1820, KL-1816 and KL-1805. Therefore, these results are very promising, where hybrids showed significant reduction in crude fibre, lower silicon content and improved nutrient quality in pasture biomass (Table 4).

The most important results were that six hybrids showed a reduction in ADF content, a high negative heterosis effect and strongest overdominance and negative heterosis for ADL content in all hybrids (Table 4). The reduced content of ADF and ADL is an important result because the biological process of lignification is assessed as the main factor limiting the nutritional value of feed and inhibiting the digestibility of dry matter [22].

### 4.9. Correlation Analysis

In the current study, the average negative correlation between the content of crude protein with NDF (*r* = −0.40) and ADF (*r* = −0.52) (Table 5) was similar to an earlier published report [74]. However, other authors defined a stronger and closer correlation of crude protein with NDF (*r* = −0.7), and with ADF (*r* = −0.64) [75], which perhaps relates to the different genetic material and experiments.

According to our data, the correlation between NDF and ADF content was high (*r* = 0.78) with a strong linear relationship. At the same time, even stronger correlations (*r* = 0.93) were reported for NDF and ADF in other forage grasses, as well as high correlations of crude fibre with NDF (*r* = 0.60), ADF (*r* = 0.83), ADL (*r* = 0.57), and with cellulose (*r* = 0.81) [76]. Similar results were found in the current study for positive but slightly lower correlations between crude fibre and NDF (*r* = 0.47), and crude fibre and ADF (*r* = 0.57) (Table 5). In the overview of the presented results, the trend shown in the correlation analysis was in the preferred direction to identify prospective genotypes.

### 4.10. Future Perspectives

In future research, the best identified hybrid lines will undergo further evaluation in forage breeding programmes. The most promising genotypes will be included in the breeding process as sources of low silicon and high pasture biomass quality. Molecular analysis of genes encoding silicon transporters in various genotypes of *P. juncea* and hybrid breeding lines will provide a key to a better understanding of the mechanism of silicon accumulation in plants and its possible association with improved pasture biomass quality.

## 5. Conclusions

In the current study, a germplasm collection with 72 *P. juncea* accessions was evaluated for pasture biomass quality traits, where six genotypes with low silicon were identified. These were used as parents, and new hybrids with low silicon and high nutrients in forage biomass and with the desired level of heterosis were produced. Further research will be carried out to understand the genetic basis of silicon transport and accumulation in *P. juncea* leaves. Overall, the presented results identified genotypes of *P. juncea* and selected hybrid combinations with low silicon content and better nutrient value of pasture biomass in plants grown in the conditions of Northern Kazakhstan.

## Figures and Tables

**Table 1:** Data for precipitation and temperature for the five main months of active plant growth in 2024 and 2025 in Shortandy (Kazakhstan), and in comparison to the average total over past years for the same period, shown as a percentage.

Year	April	May	June	July	August	Total	Average Past	% to Aver. Past
**Precipitation (mm)**								
2024	10.7	76.9	62.3	63.3	106.6	319.8	149.1	214.4
2025	1.7	8.4	26.9	11.4	35.2	83.6	149.1	56.1
**Temperature (°C)**								
2024	7.4	11.2	22.6	21.7	17.3	80.2	71.5	112.2
2025	10.7	17.5	20.5	20.7	18.1	87.5	71.5	122.4

**Table 2:** Silicon content and nutritional feed quality traits of pasture biomass in 72 accessions of *Psathyrostachys juncea* and their distribution during cluster analyses, Northern Kazakhstan, 2024–2025. Abbreviations: NDF, neutral detergent fibre; ADF, acid detergent fibre; ADL, acid detergent lignin. Significant differences for each trait between two clusters were calculated using *t*-test and designated by asterisks (* *p* < 0.05; ** *p* < 0.01; and *** *p* < 0.001), or ‘^ns^’, non-significant.

Compound Content %	Average	Range		Clusters, Feed Quality	
		Minim.	Maxim.	A (*n* = 40)	B (*n* = 32)
Silicon	2.59	1.45	4.11	2.77 ± 0.08 ***	2.37 ± 0.08 ***
Crude protein	20.71	14.84	23.69	20.34 ± 0.21 *	21.16 ± 0.29 *
Crude fibre	24.09	21.98	27.37	24.49 ± 0.20 **	23.63 ± 0.30 **
Crude ash	10.68	8.52	12.99	10.84 ± 0.11 ^ns^	10.58 ± 0.14 ^ns^
Crude fat	2.64	2.16	3.15	2.66 ± 0.04 ^ns^	2.62 ± 0.05 ^ns^
NDF	46.05	41.73	50.98	46.60 ± 0.31 *	45.41 ± 0.49 *
ADF	21.95	19.07	28.38	22.30 ± 0.18 ^ns^	21.69 ± 0.45 ^ns^
ADL	2.68	1.63	5.13	2.61 ± 0.10 ^ns^	2.70 ± 0.17 ^ns^
Hemicellulose	23.99	20.54	27.52	24.29 ± 0.24 *	23.64 ± 0.27 *
Cellulose	19.46	16.01	24.95	19.53 ± 0.29 ^ns^	19.27 ± 0.32 ^ns^

**Table 3:** Best six selected accessions of *P. juncea* for low silicon content in 2024 and 2025. The standard cultivar was used for comparison. Significant differences between groups of six accessions and the standard were calculated using *t*-test (*n* = 3 for each genotype) and designated by asterisks (*** *p* < 0.001) and ‘^ns^’, non-significant differences.

Cluster Order	Accession ID	Origin	Silicon in the First Cut, %		
			2024	2025	Average
B1	K-43934, p-0230	Kazakhstan	1.46	1.44	1.45
B2	K-46752, p-0242	Kazakhstan	1.45	1.47	1.46
B3	K-36812, p-0226	Kazakhstan	2.00	1.72	1.86
B4	K-46754, p-0241	Kazakhstan	1.60	1.58	1.59
B5	K-1731, PI-502577	Russia	1.85	1.87	1.86
B8	K-40193, p-0228	Kazakhstan	1.95	2.17	2.06
Average for 6 accessions			1.72 ^ns^	1.71 ***	1.71 ***
A1	Shortandinsky (Standard)	Kazakhstan	1.77 ^ns^	2.96 ***	2.37 ***

**Table 4:** Data for five trait measurements, including silicon content and main traits of pasture quality, estimates of true heterosis (H_true_) and degree of dominance (Hp), in parents and hybrids of *P. juncea*, presented in three groups: (**A**) reciprocal hybrids K-46752 × K-43934; (**B**) reciprocal hybrids K-43934 × K-40193; (**C**) three other hybrids with parent K-43934. Abbreviations: ADF, acid detergent fibre and ADL, acid detergent lignin. For statistical analyses of parents and hybrids, data for the best corresponding parents were underlined. Significant differences between hybrids and the underlined parent were designated by asterisks (* *p* < 0.05; ** *p* < 0.01; and *** *p* < 0.001) using *t*-test (*n* = 3). Non-significant differences were not indicated.

Parent and Hybrid ID	Cross	Silicon		Crude Protein		Crude Fibre		ADF		ADL	
(**A**)											
**Parents**											
K-46752	-	1.46 ± 0.21		20.32 ± 0.84		27.36 ± 0.92		27.65 ± 0.96		5.13 ± 0.17	
K-43934	-	1.45 ± 0.31		17.10 ± 0.97		27.21 ± 0.64		25.97 ± 0.32		4.57 ± 0.16	
**Hybrids**											
KL-1804	♀ K-46752 × ♂ K-43934	1.89 ± 0.24		19.03 ± 0.65		27.23 ± 0.81		21.70 ± 0.62 ***		1.06 ± 0.12 ***	
KL-1805	♀ K-43934 × ♂ K-46752	1.67 ± 0.22		20.65 ± 0.72		26.30 ± 0.46		21.94 ± 0.45 ***		1.34 ± 0.14 ***	
**Heterosis**		H_true_	Hp	H_true_	Hp	H_true_	Hp	H_true_	Hp	H_true_	Hp
KL-1804	♀ K-46752 × ♂ K-43934	30.3	43.0	−6.35	0.42	0.07	0.71	−16.4	6.1	−76.8	13.53
KL-1805	♀ K-43934 × ♂ K-46752	15.17	21.0	−1.62	1.14	−3.34	14.0	−15.5	5.7	−70.67	12.53
(**B**)											
**Parents**											
K-43934	-	1.45 ± 0.31		17.10 ± 0.97		27.21 ± 0.64		25.97 ± 0.32		4.57 ± 0.16	
K-40193	-	2.06 ± 0.14		21.29 ± 1.12		23.89 ± 1.32		28.38 ± 0.73		3.41 ± 0.24	
**Hybrids**											
KL-1808	♀ K-43934 × ♂ K-40193	2.11 ± 0.23		21.61 ± 0.79		24.26 ± 0.85		21.10 ± 0.58 ***		1.16 ± 0.27 ***	
KL-1809	♀ K-40193 × ♂ K-43934	1.77 ± 0.16		20.32 ± 0.66		24.20 ± 0.91		22.66 ± 0.87 **		0.74 ± 0.19 ***	
**Heterosis**		H_true_	Hp	H_true_	Hp	H_true_	Hp	H_true_	Hp	H_true_	Hp
KL-1808	♀ K-43934 × ♂ K-40193	45.5	−1.20	1.50	1.15	1.54	0.77	−18.8	5.05	−65.98	4.82
KL-1809	♀ K-40193 × ♂ K-43934	2.20	−0.07	1.15	0.53	1.29	0.79	−14.3	3.80	−78.2	5.60
(**C**)											
**Parents**											
K-43934	-	1.45 ± 0.31		17.10 ± 0.97		27.21 ± 0.64		25.97 ± 0.32		4.57 ± 0.16	
K-46754	-	1.59 ± 0.27		14.84 ± 1.27		26.52 ± 1.27		26.59 ± 1.07		4.92 ± 0.25	
K-36812	-	1.86 ± 0.13		20.32 ± 1.01		25.00 ± 1.76		25.89 ± 0.61		4.19 ± 0.22	
Shortand.	-	2.37 ± 0.34		21.75 ± 0.65		23.78 ± 1.82		22.59 ± 0.78		3.06 ± 0.10	
**Hybrids**											
KL-1816	♀ K-43934 × ♂ K-46754	2.33 ± 0.21 *		15.96 ± 1.06		25.90 ± 0.77		22.44 ± 0.32 **		2.76 ± 0.23 ***	
KL-1820	♀ K-43934 × ♂ K-36812	2.02 ± 0.16 *		18.39 ± 0.95		24.93 ± 0.64		23.68 ± 0.81 *		2.46 ± 0.31 ***	
KL-1813	♀ K-43934 × ♂ Shortand.	2.18 ± 0.19 *		22.90 ± 0.74		25.06 ± 0.89		24.88 ± 0.77 *		1.48 ± 0.24 ***	
**Heterosis**		H_true_	Hp	H_true_	Hp	H_true_	Hp	H_true_	Hp	H_true_	Hp
KL-1816	♀ K-43934 × ♂ K-46754	60.6	−11.6	−6.7	−0.008	−2.30	3.20	−13.6	12.2	−39.6	11.6
KL-1820	♀ K-43934 × ♂ K-36812	39.3	−1.85	−0.09	−0.19	−0.40	1.09	−8.5	56.2	−41.2	10.10
KL-1813	♀ K-43934 × ♂ Shortand.	50.3	−0.59	5.28	1.49	5.30	−0.80	10.13	−0.35	−51.63	3.2

**Table 5:** Correlation analysis between studied traits. Significant correlations were indicated by asterisks (* *p* < 0.05) and ‘^ns^’, non-significant. Abbreviations: NDF, neutral detergent fibre; ADF, acid detergent fibre; ADL, acid detergent lignin.

	Silicon	Crude Protein	Crude Fibre	Crude Fat	Crude Ash	NDF	ADF	ADL	Hemicellulose	Cellulose
Silicon	1									
Crude protein	0.06 ^ns^	1								
Crude fibre	−0.19 ^ns^	−0.71 *	1							
Crude fat	−0.09 ^ns^	−0.09 ^ns^	0.32 *	1						
Crude ash	0.63 *	0.04 ^ns^	−0.15 ^ns^	0.06 ^ns^	1					
NDF	−0.27 *	−0.40 *	0.47 *	0.09 ^ns^	−0.39 *	1				
ADF	−0.28 *	−0.52 *	0.57 *	0.18 ^ns^	−0.40 *	0.78 *	1			
ADL	−0.19 ^ns^	−0.25 ^ns^	0.16 ^ns^	−0.38 *	−0.20 ^ns^	0.26 *	0.41 *	1		
Hemicellulose	0.01 ^ns^	−0.13 ^ns^	−0.05 ^ns^	−0.01 ^ns^	−0.12 ^ns^	0.54 *	−0.07 ^ns^	−0.19 ^ns^	1	
Cellulose	−0.09 ^ns^	−0.23 ^ns^	0.28 *	0.04 ^ns^	0.01 ^ns^	0.17 ^ns^	0.20 ^ns^	0.04 ^ns^	0.01 ^ns^	1

## Data Availability

The original data presented in this study are included in the research paper and in the Supplementary Materials. Further inquiries can be directed to the corresponding authors.

## Supplementary Materials

The following supporting information can be downloaded at https://www.mdpi.com/article/10.3390/life16040562/s1. Table S1: List of 72 *Psathyrostachys juncea* accessions with their origin used in the study; Table S2: Distribution of 72 *P. juncea* accessions in two clusters with data for silicon and nutritional feed quality traits in this study.
